# Genomic and phenotypic characterization of *Burkholderia* isolates from the potable water system of the International Space Station

**DOI:** 10.1371/journal.pone.0227152

**Published:** 2020-02-19

**Authors:** Aubrie O’Rourke, Michael D. Lee, William C. Nierman, R. Craig Everroad, Chris L. Dupont

**Affiliations:** 1 J. Craig Venter Institute, San Diego, CA, United States of America; 2 Exobiology Branch, NASA Ames Research Center, Mountain View, CA, United States of America; 3 Blue Marble Space Institute of Science, Seattle, WA, United States of America; University of Minnesota, UNITED STATES

## Abstract

The opportunistic pathogens *Burkholderia cepacia* and *Burkholderia contaminans*, both genomovars of the *Burkholderia cepacia* complex (BCC), are frequently cultured from the potable water dispenser (PWD) of the International Space Station (ISS). Here, we sequenced the genomes and conducted phenotypic assays to characterize these *Burkholderia* isolates. All recovered isolates of the two species fall within monophyletic clades based on phylogenomic trees of conserved single-copy core genes. Within species, the ISS-derived isolates all demonstrate greater than 99% average nucleotide identity (with 95–99% of genomes aligning) and share around 90% of the identified gene clusters from a pangenomic analysis–suggesting that the two groups are each composed of highly similar genomic lineages and their members may have all stemmed from the same two founding populations. The differences that can be observed between the recovered isolates at the pangenomic level are primarily located within putative plasmids. Phenotypically, macrophage intracellularization and lysis occurred at generally similar rates between all ISS-derived isolates, as well as with their respective type-terrestrial strain references. All ISS-derived isolates exhibited antibiotic sensitivity similar to that of the terrestrial reference strains, and minimal differences between isolates were observed. With a few exceptions, biofilm formation rates were generally consistent across each species. And lastly, though isolation date does not necessarily provide any insight into how long a given isolate had been aboard the ISS, none of the assayed physiology correlated with either date of isolation or distances based on nucleotide variation. Overall, we find that while the populations of *Burkholderia* present in the ISS PWS each maintain virulence, they are likely are not more virulent than those that might be encountered on planet and remain susceptible to clinically used antibiotics.

## Introduction

**Fig 1:**
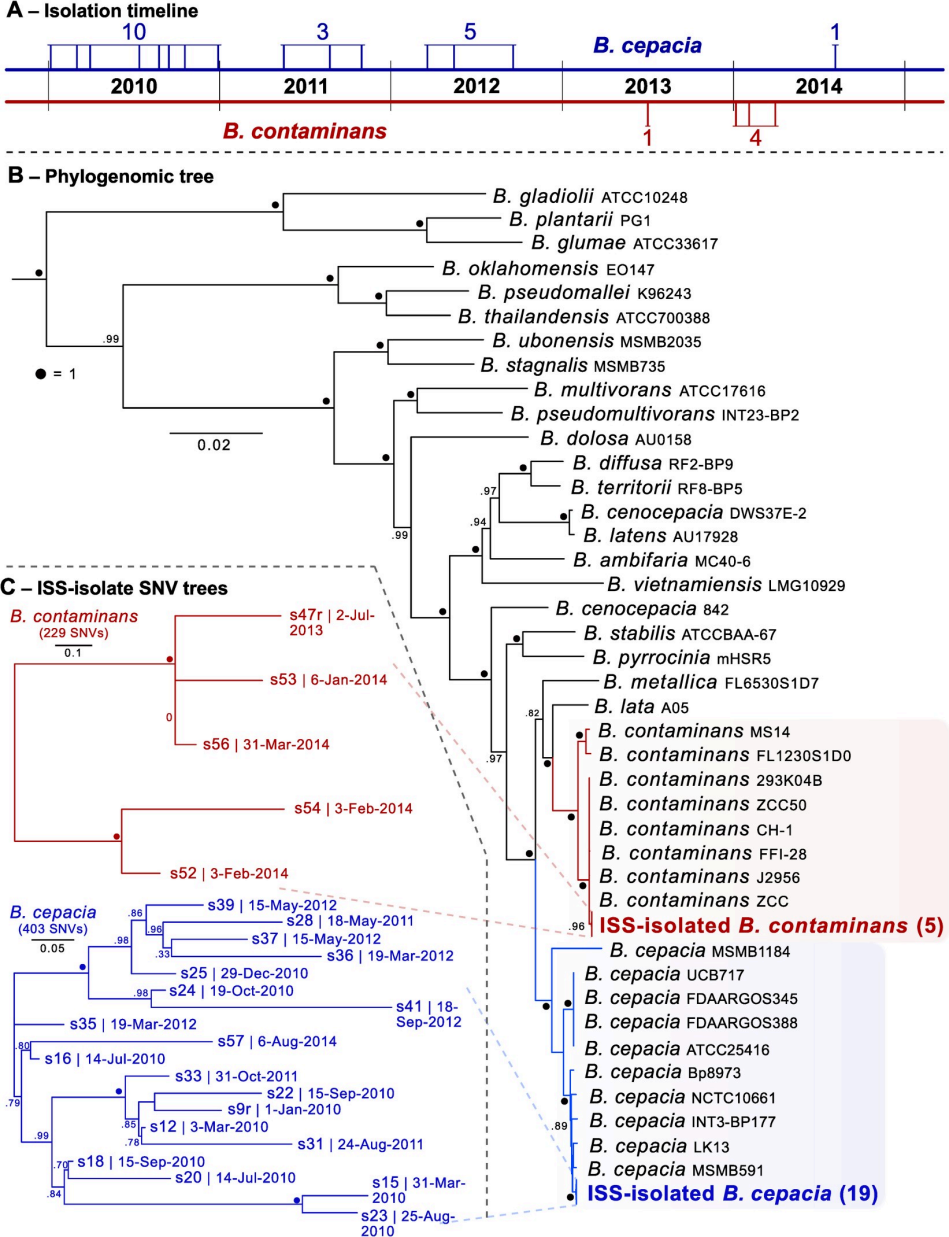
Isolate overview. **(A)** Isolation timeline (dates with multiple isolates a represented by a single line). **(B)** Estimated maximum-likelihood phylogenomic tree based on aligned and concatenated amino acid sequences of 203 single-copy genes designed for targeting Betaproteobacteria–rooted with *Ralstonia pickettii* 12J (GCF_000020205.1). Numbers in parentheses indicate number of isolates in that clade. **(C)** ISS-derived isolate SNV trees for each *Burkholderia* species. Date of isolation follows the unique identifiers. Those with an “r” represent the genome used as the reference for that SNV tree. Total aligned proportion of all genomes used in generating the SNV tree was greater than 70% in both species.

Microbial surveillance of the surfaces, air, and potable water system (PWS) of the International Space Station (ISS) has been implemented by National Aeronautics and Space Administration (NASA) to ensure crew health within this unique closed environment. These efforts, which use standard culturing techniques, have been conducted over twelve years and 22 missions and began shortly after the potable water dispenser (PWD) was launched on STS-126 in November of 2008. On-orbit operations using the PWD began in early 2009 and continue to this day. The organisms *Burkholderia cepacia* and *B*. *contaminans*, both genomovars of the *Burkholderia cepacia* complex (BCC), have been frequently cultured from the PWD of the ISS. The isolates analyzed in the current study were collected between January 6, 2010 during mission 22 and August 6, 2014 during mission 40 (Fig 1, S1 Table). The PWS in combination with the PWD is a water recycling system that utilizes physical and chemical techniques to filter, decontaminate, and sterilize water used for drinking and food hydration [1–3]. *Burkholderia* spp. are known to withstand disinfection and sterilization procedures as they display a moderate to high-tolerance to stress such as UV-C radiation, antibiotics, and high heavy-metal concentrations [4].

*Burkholderia* spp., among other bacterial contaminants, are likely to have been introduced into the PWD during unit assembly prior to launch [1–3]. *Burkholderia* spp. are known to survive long periods in distilled water [5,6]. This ability to survive in distilled water with minimal additives has made them problematic for healthcare, as hospital-acquired BCC infections can arise from contaminated disinfectants, anesthetic solutions, distilled water, and aqueous chlorhexidine solutions [4]. The ISS PWD uses flushes of 40ppm elemental iodine for decontamination, followed by iodine removal by a deiodination filter before astronaut use [1–3]. Some *Burkholderia* isolates have been found to be resistant to iodine and can even survive in iodine solutions [7], providing pre-observed phenotypes of this lineage that may be capable of maintaining a continued presence in the ISS PWD despite disinfection efforts.

*Burkholderia* species are known to propagate in both nutrient-poor and -rich soil environments, in addition to within human host cells [8]. They are commonly isolated from soil, plant rhizospheres, and water [9], as well as from the sputum of cystic fibrosis (CF) patients with chronic infection [10]. BCC members pose a significant threat to individuals with CF (accounting for 85% of all infections in CF patients [10]) and otherwise immune-compromised patients due to an exacerbation of pulmonary infections, which can lead to morbidity and mortality [11]. The antibiotic resistance and intracellular survival capabilities of BCC members make them unamenable to many therapeutics in susceptible infected patients [12]. BCC species are nearly indistinguishable from one another by biochemical testing because all members have large genomes ranging from 6-9Mb that harbor the potential for considerable plasticity and diversity within [13]. *B*. *contaminans*, however, can be distinguished by their yellow pigmentation and hemolytic quality [13]. CF infections with this species is the most prevalent in Spain, Portugal and the Ibero-American countries, whereas *B*. *cepacia* infections are more prevalent in the Iberian Peninsula alone [14]. The genomes of BCC organisms can mutate rapidly during infections or when subjected to high-stress conditions [15], the latter of which might be satisfied by the sequential iodine treatments of the PWD. The genus is known to have several mobile genetic elements (MGEs), which can promote the transfer and acquisition of virulence and antibiotic resistance genes; BCC organisms have genomic islands, such as the *B*. *cepacia* epidemic strain marker (BCESM), containing genes linked to virulence and metabolism, quorum sensing, transcriptional regulation, fatty acid biosynthesis, and transposition [15].

The PWD unit of the ISS was assembled in a cleanroom facility and then primed on Earth using an extensive process to ensure no gas bubbles existed within the lines that could lock the apparatus upon installation in orbit. During build and delivery on planet, the system was maintained using a 20 to 30 ppm iodine and a 6% hydrogen peroxide solution flush [1]. The primed system sat dormant for 6 months before installation on the ISS [1,3]. Microbial surveillance was conducted on the system after installation and the bacterial load was 85 CFU/mL, which exceeded the 50 CFU/mL limits set for ISS potable water, leaving the sole source of water on the US module out-of-order [1], during which time the Russian system was used as a back-up. The US system was flushed with the biocide iodine (I_2_), first at what turned out to be a sub-inhibitory concentration of 4ppm, as subsequent measurements revealed an increase in the microbial load [1]. Further testing revealed that 40ppm was the necessary concentration of iodine flush to achieve the drinkable 50 CFU/mL maximum bacterial load [1]. Iodine flushes are still intermittently administered to the system after durations of PWD stagnation. Our focal *Burkholderia* species are known to survive not only in distilled water, but also in iodine solutions and resist reactive oxygen species such as hydrogen peroxide; therefore, these flushes may have reduced the overall microbial load of the PWD while inadvertently selecting for the *Burkholderia* species within the system. Here we characterize genomic and phenotypic properties of *Burkholderia* isolates obtained from the ISS PWD.

## Results and discussion

Twenty-four isolates were collected over 4.5 years from the PWD of the ISS (Fig 1A). The genomes of these 24 ISS-PWD *Burkholderia* isolates were sequenced and *de novo* assembled with SPAdes v3.12.0 ([16]; isolate and assembly summary information in S1 Table). The recovered genomes within each species were all found to have an ANI of greater than 99% with 95–99.9% alignment in all cases (fastANI v1.2; [17]), suggesting that they are each of highly similar genomic lineages and may have stemmed from the two distinct founding strains. This scenario has been observed previously in *Burkholderia cenocepacia* infections where the initial colonization of affected patients was thought to be by an individual strain that subsequently diversified once in the host [18]. This has also been observed for *Pseudomonas aeruginosa* as it adapts to the airways of cystic fibrosis patients where clones of cells experience selection which in turn accumulate genetic variants that help promote survival in the long-term [19]. An estimated maximum-likelihood phylogenetic tree based on amino acid sequences of 203 single-copy genes specific to Betaproteobacteria was generated with GToTree v1.4.4 ([20]; Fig 1B). This placed 19 of the isolates within their own monophyletic clade within the *B*. *cepacia*, and 5 within their own monophyletic clade within the *B*. *contaminans* (Fig 1B; see S1 Fig for full tree). Single-nucleotide variant (SNV) trees (Parsnp v.1.2; [21]) generated for each group of isolates revealed no clear groupings based on time of isolation (Fig 1C; Mantel test *p*-value = 0.2). The *B*. *cepacia* were isolated over the entire sampling period, while *B*. *contaminans* were only isolated during 2013 and 2014 (Fig 1A).

### Pangenomic analysis

**Fig 2:**
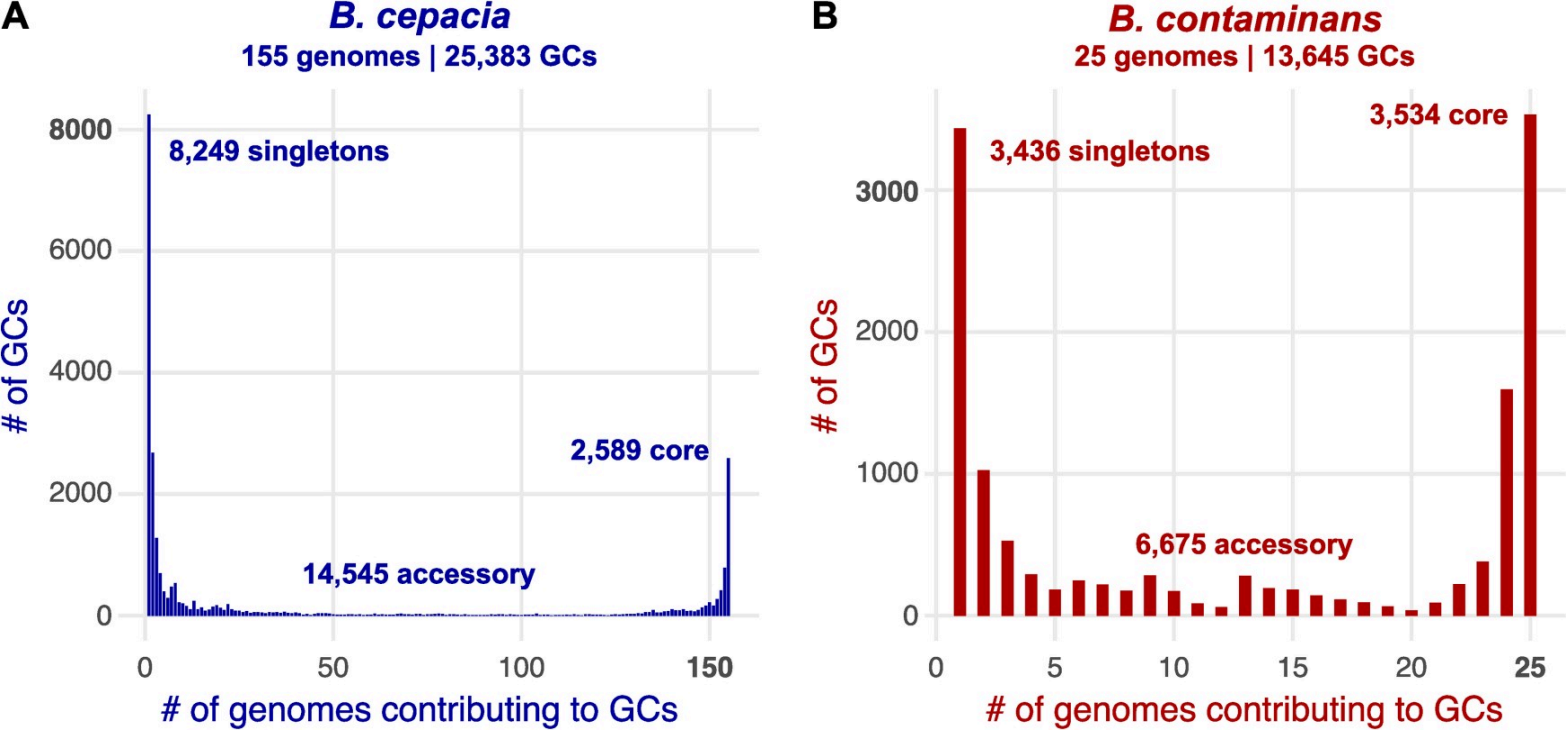
**Gene-cluster distributions** for **(A)** *B*. cepacia and **(B)** *B*. *contaminans*.

We performed a pangenomic analysis of each species first incorporating all assemblies available from NCBI (as accessed on 20-Aug-2019). Gene clusters (GCs) were identified with MCL clustering [22] as employed within anvi’o v5.5 [23]. Across the incorporated 155 *B*. *cepacia* genomes (136 reference from NCBI and 19 derived from the ISS PWS), the average gene count was 7,461 ± 479 (mean ± 1SD; summarized in Table 1). The MCL clustering approach yielded the identification of 25,383 total GCs: 2,589 with genes contributed by all (“core”; 10.2%); 8,249 with genes contributed by only single genomes (“singletons”; 32.5%); and 14,545 contributed by some mixture (“accessory”) (Fig 2A). A total of 25 *B*. *contaminans* genomes were incorporated (20 references from NCBI and 5 ISS-isolates), with an average gene count of 7,580 ± 539. From these, a total of 13,645 GCs were generated: 3,534 core (25.9%); 3,436 singletons (25.2%); and 6,675 accessory GCs (49.0%; Fig 2B; summarized in Table 1). It should be noted the disparity between the number of identified core, singleton, and accessory genes in *B*. *cepacia* versus those identified in *B*. *contaminans* at this point is not meant to convey anything about potential differences in gene-content diversity between the two species–as the number of genomes available/incorporated for each and the breadth of phylogenetic diversity spanned by the incorporated genomes of the two groups both vary greatly.

**Table 1:** *Burkholderia* pangenome overview of counts.

***B*. *cepacia***		
	With refs	ISS-isolates only
# genomes	155	19
# genes (mean ± 1 SD)	7,461 ± 479	7,762 ± 131
Total GCs	25,383	7,688
Core GCs (% of total)	2,589 (10.2%)	7,065 (91.9%)
Accessory GCs	14,545 (57.3%)	603 (7.8%)
Singleton GCs	8,249 (32.5%)	20 (0.26%)
***B*. *contaminans***		
	With refs	ISS-isolates only
# genomes	25	5
# genes (mean ± 1 SD)	**7**,580 ± 539	7,852 ± 87
Total GCs	13,645	7,778
Core GCs (% of total)	3,534 (25.9%)	7,509 (96.5%)
Accessory GCs	6,675 (49.0%)	106 (1.4%)
Singleton GCs	3,436 (25.2%)	163 (2.1%)

We scanned for functional enrichment or depletion in the ISS-derived isolates as compared to the reference genomes based on normalized frequencies of presence/absence across the two groups (see Methods). It should be kept in mind that given the phylogenetic landscape of both ISS-derived groups–with each forming monophyletic clades within their respective species (Fig 1B; S1 Fig)–any functional differences observed may be simply due to evolutionary divergence as a whole, rather than being due to their source of isolation (the ISS). Of the *B*. *contaminans* pangenome, this revealed no depleted functions (i.e. functional annotations a with signifanctly lower normalized abundance) in the ISS-derived isolates, and 8 functions enriched including those commonly associated with viruses and conjugative plasmids (Table 2; full results in S2 Table). The functions unique to the ISS-derived *B*. *contaminans* included genes annotated as Invasion protein IagB (which has been associated with *Salmonella enterica* subsp. *enterica* ser. Typhi invasion of HeLa cells [24]) and Nucleoside 2-deoxyribosyltransferase.

**Table 2:** ISS-derived *B*. *contaminans* enriched functions as compared to references.

Annotation (NCBI PGAP)	Occurrence in ISS-isolates	Occurrence in Refs	Corrected p-value
Invasion protein IagB	5/5	0/20	0.09
Nucleoside 2-deoxyribosyltransferase	5/5	0/20	0.09
PH domain-containing protein	5/5	1/20	0.09
RepA replicase	5/5	1/20	0.09
Metallophosphatase family protein	5/5	1/20	0.09
Pilus assembly protein PilN	5/5	1/20	0.09
Prepilin type IV pili	5/5	1/20	0.09
Type IV secretion system protein VirB3	5/5	1/20	0.09

In contrasting the functional annotations of the 19 ISS-derived *B*. *cepacia* with the 136 references, 265 functions were found to be enriched, with 98 found to be depleted (5 of each presented in Table 3; full results in S3 Table). Each *B*. *cepacia* isolate contained copies of genes annotated as a Toprim domain-containing protein and a 3-carboxyethylcatechol 2,3-dioxygenase that were not found detected in any of the 136 reference genomes incorporated. 3-carboxyethylcatechol 2,3-dioxygenase is known to be involved in the breakdown of polycyclic aromatic hydrocarbons (PAHs) to be used as a carbon and energy source [25]. Additionally, the ISS *B*. *cepacia* isolates show a loss of a Lipoteichoic acid (LTA) synthase family protein, that is likely related to capsule synthesis and therefore suggests an attenuation of this virulence element [26].

**Table 3:** ISS-derived *B*. *cepacia* enriched/depleted functions as compared to references.

Annotation (NCBI PGAP)	Occurrence in ISS-isolates	Occurrence in Refs	Corrected p-value
Toprim domain-containing protein	19/19	0/136	0.00
3-carboxyethylcatechol 2,3-dioxygenase	19/19	0/136	0.00
Metal transporter	19/19	1/136	0.00
Plasmid mobilization relaxosome MobC	19/19	1/136	0.00
Peptidase M4	19/19	3/136	0.00
Sugar transporter	0/19	133/136	0.00
LTA synthase family protein	0/19	132/136	0.00
Class II aldolase family protein	0/19	123/136	0.00
Nit6803 family nitriliase	0/19	111/136	0.00
MSMEG_0572 N starvation response	0/19	111/136	0.00

**Fig 3:**
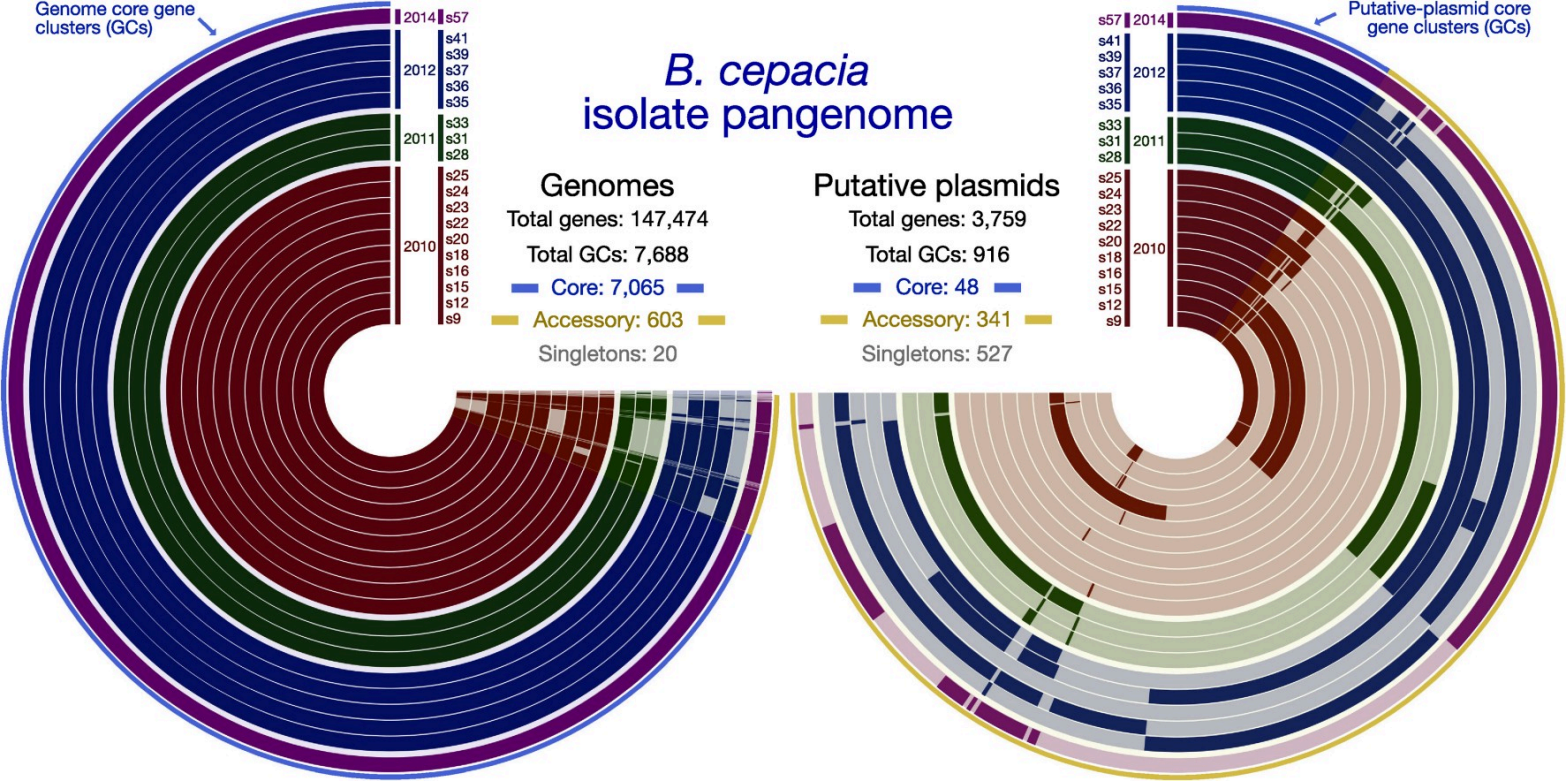
**Pangenomics visualizations of the 19 ISS *B. cepacia* isolate genome assemblies (left) and identified putative plasmids (right).** Each concentric circle radiating out from the center represents an isolate, identified at the top of each next to the year they were isolated. Wrapping around the circles are the generated gene clusters (GCs), where a solid mark for an isolate at a given GC indicates that particular isolate contributed a gene to that gene cluster, and the absence of a solid color indicates that isolate did not contribute a gene to that gene cluster. The very outer layer of each is a blue and yellow line. The blue line highlights core gene clusters. The yellow line highlights accessory GCs. The putative-plasmid pangenome on the right does not include singletons in the visualization.

As noted above, the recovered genomes within each species were all found to have an ANI of greater than 99% with 95–99.9% alignment in all cases, and each form monophyletic clades in relation to other members of their respective species (Fig 1B; S1 Fig). Pangenomic analyses incorporating solely the ISS-derived isolates revealed greater than 90% of the identified GCs having genes contributed from all genomes for both species, demonstrating the presence of highly conserved core genomes in each. For the 19 *B*. *cepacia* isolates, 7,688 total GCs were generated: 7,065 core (91.9%); only 20 singletons (0.26%); and 603 accessory GCs (7.8%; Fig 3, left side; summarized in Table 1). Of the singleton GCs with functional annotations (5/20), most annotations were present in other GCs–meaning the sequences diverged enough to not cluster together, but were similar enough to be annotated the same way. The one exception was annotated by NCBI as an autotransporter domain-containing protein (from isolate s20, gene ID D7204_40525). For the 5 *B*. *contaminans* isolates, 7,778 GCs were generated: 7,509 core (96.5%); 163 singletons (2.1%); and 106 accessory GCs (1.4%; summarized in Table 1). Of the 163 singleton GCs from *B*. *contaminans*, 46 had annotations associated with them. Twenty-eight of these annotations were found in other GCs, while 18 were only annotated in one genome. Interestingly, 17 of these 18 all came from the same isolate s47, which was the first *B*. *contaminans* isolate recovered from the ISS (Fig 1C); these functional annotations are presented in Table 4. All gene calls, amino acid sequences, and annotations are available in S4 Table. Clustering based on GCs did not recapitulate the SNV trees (S2 and S3 Figs).

**Table 4:** ISS-derived *B*. *contaminans* singleton-GC functional annotations.

Annotation (NCBI PGAP)	Isolate	Gene ID
Nickel resistance protein	s47	D7S92_31485
Conjugal transfer protein TrbE	s47	D7S92_31155
Conjugal transfer protein TrbI	s47	D7S92_31155
DUF1259 domain-containing protein	s47	D7S92_31415
C-type cytochrome biogenesis protein CcsB (ccsB)	s47	D7S92_31465
Type IV secretion system family protein	s47	D7S92_31160
Toprim domain-containing protein	s47	D7S92_35590
P-type DNA transfer ATPase VirB11 (virB11)	s47	D7S92_31190
Replication initiator protein A	s47	D7S92_31330
Type II secretory pathway component PulF-like protein	s47	D7S92_31550
Chromate resistance protein	s47	D7S92_31420
Type IV secretion system protein VirD4	s47	D7S92_31125
Type IV secretion system protein VirB8	s47	D7S92_31175
Zinc metalloproteinase Mpr protein	s47	D7S92_31335
Zeta toxin family protein	s47	D7S92_31320
Nickel/cobalt efflux protein RcnA	s47	D7S92_31490
Conjugative relaxase	s47	D7S92_31130
Type IV secretion protein Rhs	s56	D7S91_34560

### Plasmid analysis

Putative plasmids were computationally identified in the ISS-derived isolates using plasmidSPAdes [27], which operates largely based on coverage and searching the assembly graph for putative circular subgraphs. This process identified putative plasmid contigs from each of the 19 *B*. *cepacia* isolates and 2 of the 5 *B*. *contaminans* isolates (s47 and s52; annotations and sequences for all can be found in S5 Table. Running a pangenomic analysis on the 19 *B*. *cepacia* putative plasmids generated a total of 916 GCs: 48 core (5.2%); 527 singletons (57.5%); and 341 accessory GCs (37.2%; Fig 3, right side). Despite the high similarity between the ISS genomes as a whole, the majority of variability that does exist within their genetic complement (Fig 3, left side ≈3–3:30 “o’clock”) appears to be found–and maintained through time–among the putative plasmids that were identified (Fig 3, right side). The 2 recovered putative plasmids from *B*. *contaminans* had little overlap based on GCs. With a total of 475 GCs, only 3 had genes contributed from both genomes (0.6%), leaving the remaining 472 as singletons. Annotations from NCBI’s Clusters of Orthologous Genes (GOGs; [28]) of coding sequences from the putative plasmids reveal elements typical of conjugative plasmids, such as DNA replication proteins (DnaC), plasmid stabilization proteins (ParE), and Type IV secretion system components (T4SS; Table 5). Additionally, *B*. *cepacia* putative plasmids contained a catechol 2,3-dioxygenase, which serves as a contamination indicator as it is able to breakdown polycyclic aromatic hydrocarbons [29,30], and lysophospholipase, an enzyme known to be used by ingested bacteria to avoid phagocytosis by macrophage [31]. Despite containing this small conserved core, the larger plasmids in particular contain multiple copies of genes with annotations such as bacteriophage DNA transposition protein, AAA+ family ATPase (s9, s36, s39, s57), Virulence-associated protein-VagC (virulence associated gene C; s16, s28, s35, s36, s39), and additional elements of the T4SS, VirB8 (S5 Table). We additionally see transposase-related genes in the *B*. *cepacia* isolates with a mean copy number per putative plasmid of 17.57 ± 5.7 (mean ± 1 SD), and an integrase element with a mean copy number of 2.47 ± 0.51, suggesting DNA rearrangements and duplications among the conjugative plasmids of ISS *B*. *cepacia* may be a result of these mobile genetic elements.

**Table 5:** COG functional annotations and copy numbers per putative plasmid for *B*. *cepacia* and *B*. *contaminans*.

***B*. *cepacia* putative-plasmid annotations (19)**	
**Annotation (COG)**	**Mean copy # ± 1 SD**
Plasmid stabilization system protein ParE	1 ± 0
Site-specific DNA-cytosine methylase	1 ± 0
Type IV secretory pathway, component VirB8	1 ± 0
Lysophospholipase	1.11 ± 0.5
Catechol 2,3-dioxygenase	1.84 ± 1.1
Arsenite efflux pump ArsB, ACR3 family	1.89 ± 1.1
Integrase	2.47 ± 0.5
DNA replication protein DnaC	3.05 ± 0.2
Cu/Ag efflux pump CusA	3.05 ± 3.4
DNA-binding transcriptional regulator, LysR family	3.11 ± 5.9
Transposase-related	17.11 ± 5.7
***B*. *contaminans* putative-plasmid annotations (2)**	
**Annotation (COG)**	**Mean copy # ± 1 SD**
Cellulose biosynthesis protein BcsQ	1 ± 0
mRNA-degrading endonuclease, toxin component of the MazEF toxin-antitoxin module	1 ± 0
Tfp pilus assembly protein PilT, pilus retraction ATPase	1 ± 0
Type II secretory pathway component GspD/PulD (secretin)	1.5 ± 2.1
Type IV secretory pathway, component VirB8	1.5 ± 2.1
Type IV secretory pathway, VirB9 components	2 ± 1.4
Chromosome segregation protein ParB	2 ± 0
Chromate transport protein ChrA	2 ± 0
DNA-binding protein H-NS	2.5 ± 0.7
Soluble lytic murein transglycosylase	3 + 2.8
Transposase-related	5 ± 5.7

As for *B*. *contaminans*, in addition to harboring elements of both the T4SS and Type II secretion system (T2SS), the plasmids have a soluble lytic murein transglycosylase harboring a putative invasion domain LysM at a mean copy number of 3 ± 2.8. The lytic transglycosylase, LtgG, has recently reported for its role to control cell morphology and virulence in *Burkholderia pseudomallei* [32]. Furthermore, we see each harboring a copy of the toxin component of the MazEF toxin-antitoxin system suggesting these ISS *B*. *contaminans* isolates may be able to control their transition to the dormant persister state [33]. Again, we see transposase-related genes with a mean copy number of 5 ± 5.7.

#### *B*. *cepacia* plasmid encoded features

The largely coverage-based plasmidSPAdes [27] approach identified putative plasmids in all 19 of the ISS *B*. *cepacia* isolates. A pangenomic view of these putative plasmids for *B*. *cepacia* revealed a conserved functional core of elements including the T4SS VirD4 gene as well as a catechol 2,3-dioxygenase and lysophospholipase (Table 5). As with *B*. *contaminans*, *B*. *cepacia* harbor elements of the Type IV secretion system (T4SS) on putative plasmids as well as an enrichment of transposase-related genes (S5 Table). The observation that, within *B*. *cepacia*, transposase-related genes are present at a mean copy number of 17.57 ± 5.7 per putative-plasmid, along with an integrase element with mean copy number of 2.47 ± 0.51, provides a possible beneficial mechanism to be used by the bacteria when the population is under stress via promoting DNA rearrangement and duplications [34]. T4SS mediate horizontal gene transfer, which contributes to genomic plasticity and possibly the evolution of pathogens through the spread of antibiotic resistance or virulence genes [35]. They are multi-subunit, cell-envelope-spanning structures consisting of a pilus and a secretion channel whereby DNA or protein is translocated outside of the cell to either another bacterial or host cell or to the surrounding environment. Furthermore, lysophospholipase is an enzyme that frees fatty acids from lysophospholipids (LPLs) and in turn generates cytotoxic LPLs. As a result, LPLs are considered to be virulence factors of bacteria as they are found to help bacteria escape phagosomes in host cells after a few rounds of intracellular multiplication [31]. The cytotoxicity they generate allows the bacteria to rupture out of a macrophage or epithelial cell, and in addition, destroy lung surfactant and generate signal transducers such as lysophosphatidylcholine, which in turn can induce inflammation [31]. Therefore, their plasmid-encoded genetic content suggests the potential for rapid adaptation, infection and cytotoxicity from these isolates.

Also found as a core gene in each plasmid is a catechol 2,3-dioxygenase (C23O). The presence of such C23Os is commonly a water quality indicator [36]. C23Os degrade polycyclic aromatic hydrocarbons (PAHs) such as benzene, toluene, ethylbenzene, and xylenes, as well the degreaser and common groundwater contaminant trichloroethylene (TCE). Toluene has been identified among the organic compounds found in the humidity condensate samples from the US Space Shuttle cabin [37]. The Shuttle did not use a water reclamation system, but the ISS does, as it reclaims water from urine and urine flush water, humidity condensate, personal hygiene water, and effluent from the crew health care systems. On a 1994 Shuttle mission, bags used to store urine samples for a Life Sciences experiment were giving off strong odors, and a post-flight assessment attributed this to the presence of volatile microbial metabolites which included 1,1,1-trichloroethane and toluene [38]. In addition, trichloroethene (TCE) was found in one of two samples processed from the galley cold-water ports on Mir-21 [38] in the range of 1.8–2.3 ug/L–the EPA has set a maximum contaminant level (MCL) of 5μg/L (5 ppb) in drinking water for TCE (U.S. Environmental Protection Agency 1985). Therefore, in the event that trace amounts of PAHs are present in the PWD, *Burkholderia* species maybe using C23Os to catabolize the compounds into usable carbon sources in order to facilitate their survival in the low-nutrient environment of the PWD of the ISS.

One potential selective pressure these isolates are exposed to is the intermittent dose of iodine which is used to disinfect the PWD. Alternatives to iodine have been considered for future PWD microbial disinfection, such as the use of silver in the form of silver (I) fluoride [3,39]. The effect of silver treatment upon *B*. *cepacia* has been explored as a clinical alternative to antibiotics [40] and will likely reduce this microbial load. However, we find that the ISS *B*. *cepacia* isolates analyzed in this study all harbored a Cu/Ag efflux pump (CusA) at a mean copy number of 3.05 ± 3.4 on a conjugative plasmid (Table 5). Conjugative plasmids can be shared and could share the ability to pump silver out of an affected cell. Experimental designs testing the effects of the iodine disinfection process, and possibly how silver treatments may compare, would be of value moving forward. Our next steps would be to generate a transposon mutant library from an ISS isolate and subject the library to iodine and silver (I) fluoride treatments to investigate which genes may aid in survivorship.

#### *B*. *contaminans* plasmid encoded features

Putative plasmids were also identified in 2 of the 5 ISS *B*. *contaminans* isolates (s47 and s52). Both possess annotated T4SS and T2SS components as well as a soluble lytic murein transglycosylase harboring a putative invasion domain LysM at a mean copy number of 3 ± 2.83 (Table 5). The lytic transglycosylase, LtgG, has recently reported for its role to control cell morphology in *Burkholderia pseudomallei* and its virulence in the BALB-C mouse model [32]. Both also harbor a copy of the toxin component of the MazEF toxin-antitoxin system, suggesting the possibility for these ISS *B*. *contaminans* isolates to control their transition to the dormant persister state. Toxin-antitoxin systems are known to contribute to cell dormancy by halting cellular machinery through use of the toxin that is counteracted by the antitoxin once the stressor is removed [33] and such a scenario is a possible explanation here. And as with *B*. *cepacia*, we also see a high-copy number of transposase-related genes (5 ± 5.7; mean ± 1SD).

### Phenotypic assessment of ISS *Burkholderia* species

#### Macrophage infection assay

**Fig 4:**
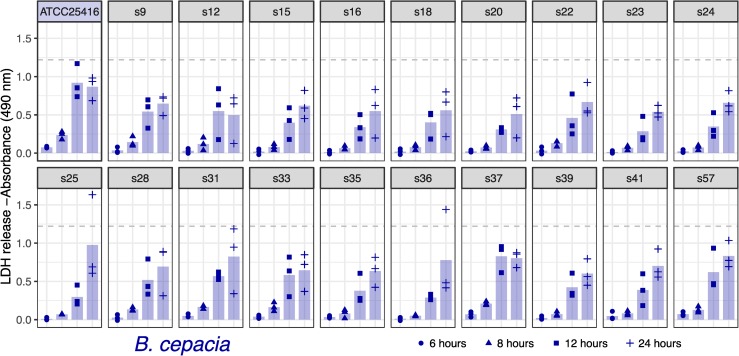
*B*. *cepacia* lactase dehydrogenase (LDH) release from macrophage. 6, 8, 12, and 24 hours post exposure to *B*. *cepacia* along with terrestrial reference strain ATCC25416. Increased absorbance correlates to increased LDH release from macrophage. Horizontal dotted grey line indicates “TritonX” complete lysis. As compared to the reference strain via two-sided t-tests, none were found to have an adjusted p-value < 0.61 (adjusted by Bonferroni correction). Bars depict means, all points are plotted (n = 3).

**Fig 5:**
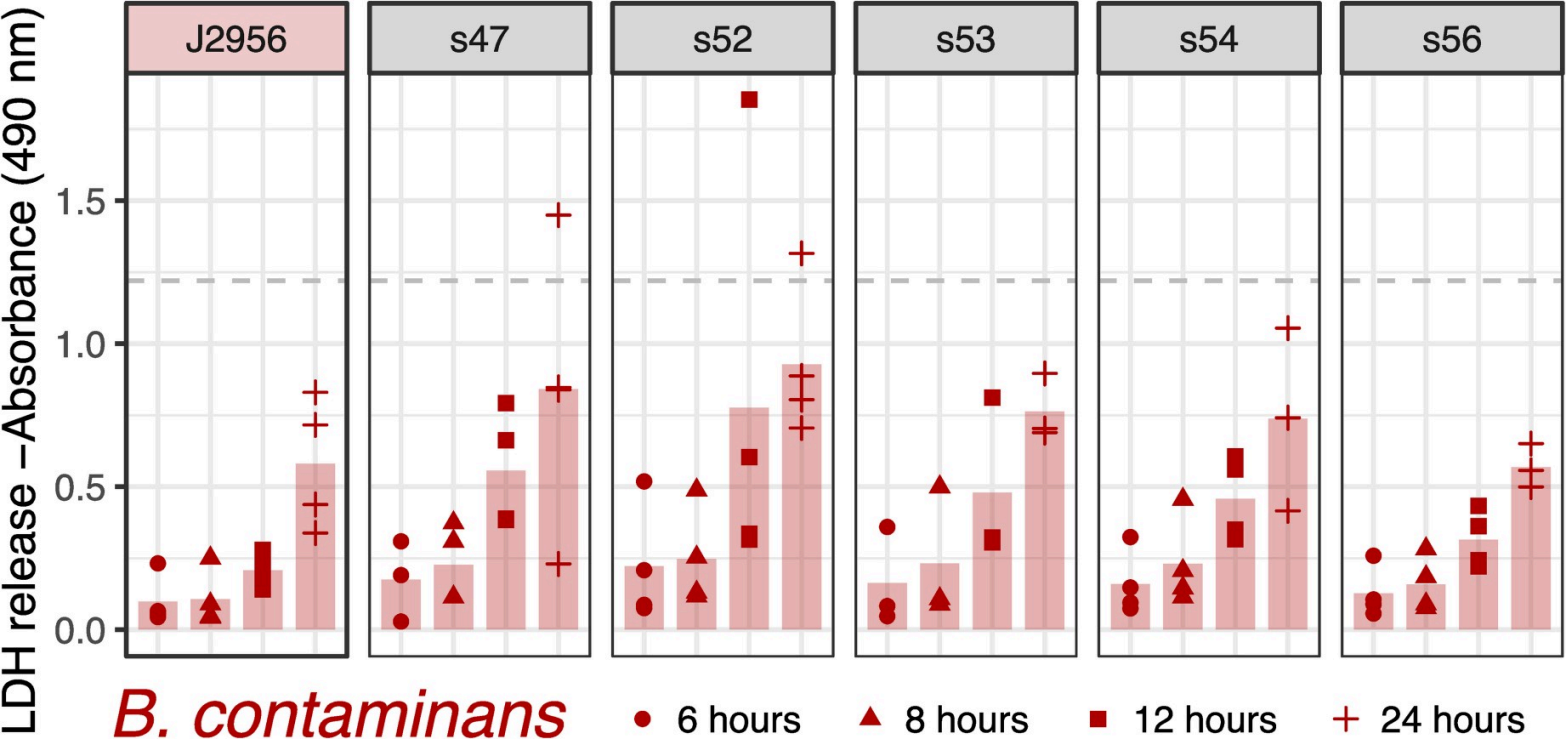
*B*. *contaminans* lactase dehydrogenase (LDH) release from macrophage. Same as Fig 4, but here for *B*. *contaminans* with terrestrial reference strain J2956. As compared to the reference strain via two-sided t-tests, none were found to have an adjusted p-value < 0.68 (adjusted by Bonferonni correction).

**Fig 6:**
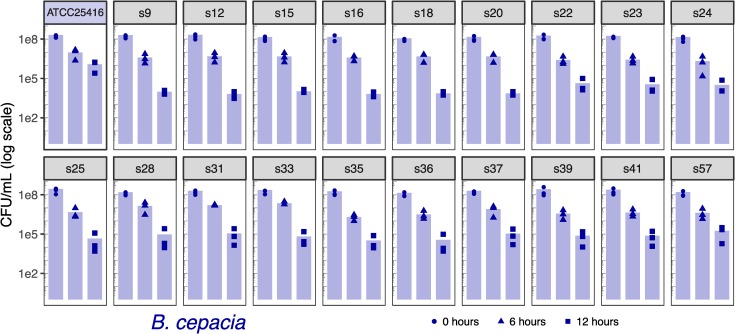
*B*. *cepacia* colony forming units per milliliter (CFU/mL) following addition of J774A.1 mouse macrophage. Hours indicate time from addition. Decreased counts are assumed to be due to the cells’ increased propensity for macrophage lysis over time. As compared to the reference strain via two-sided t-tests, none were found to have an adjusted p-value < 1 (adjusted by Bonferonni correction). Bars depict means, all points are plotted (n = 3).

**Fig 7:**
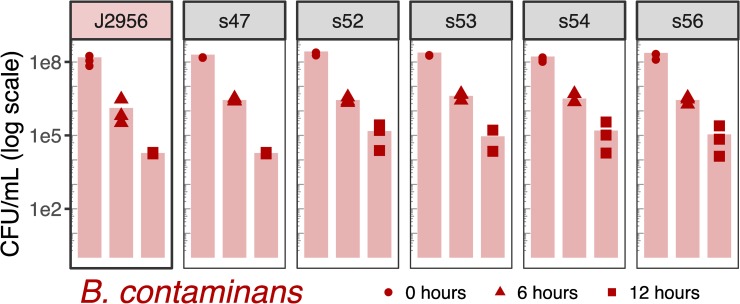
*B*. *contaminans* colony forming units per milliliter (CFU/mL) following addition of J774A.1 mouse macrophage. Same as Fig 6, but here for *B*. *contaminans*. As compared to the reference strain via two-sided t-tests, none were found to have an adjusted p-value < 0.99 (adjusted by Bonferonni correction).

The identification of putative virulence mechanisms within the ISS *Burkholderia* genomes led us to explore the ability for the ISS *B*. *cepacia* and *B*. *contaminans* isolates to invade and persist within macrophage in a cell culture. For reference, we have included two terrestrial strains from the same lineages (*B*. *cepacia* ATCC25416 and *B*. *contaminans* J2956). We note that while these are not “controls” as the ISS isolates were collected over time with no experimental design in place, and the source of these reference strains being terrestrially derived versus being derived from the ISS covaries with phylogenetic divergence from the ISS isolates, we include them as type strain references. We used two metrics for this assessment: 1) the ability of the bacteria to invade macrophage cells and be retained intracellularly, which allows for the bacteria to replicate without lysosomal degradation in turn lending to chronic infections; and 2) the ability for the bacteria to cause macrophage cell lysis as assessed by the amount of lactate dehydrogenase (LDH) released by the cell. Such cell lysis in turn can trigger cytokine release and an inflammatory response. We present the cell counts for the intracellularized bacteria and LDH data for all ISS-derived isolates and *B*. *contaminans* J2956 and *B*. *cepacia* ATCC25416 reference strains. Macrophage cell lysis was quantified by the amount of LDH released in the cell culture media at six, eight, twelve and 24 hours post-inoculation (Figs 4 and 5). Bacteria that were internalized by macrophage were quantified and reported in colony forming units per milliliter (CFU/mL) measured at six and twelve hours post-inoculation (Figs 6 and 7).

Across all timepoints, no significant differences were detected between *B*. *cepa*cia and its reference strain (minimum adj. p-value = 1; Fig 4) or *B*. *contaminans* and its reference (minimum adj. p-value = 0.68; t-tests followed by Bonferroni correction; Fig 5). At six and eight hours post infection, relatively little LDH-release was observed for both the ISS-derived *B*. *cepacia* isolates and terrestrial reference strain (Fig 4). The *B*. *contaminans* ISS-derived isolates appear to be triggering cell lysis at generally similar rates to the terrestrial reference, though with high variability across the experimental triplicates (Fig 5). At twelve hours post-infection, the lysis trend exhibited for each isolate exists but at twice that of the magnitude of LDH released at eight hours post infection. When comparing only the ISS-derived isolates to each other, an ANOVA of the *B*. *cepacia* LDH data by timepoint yielded a difference in means of the isolates for the 8-hour timepoint (*p-*value = 4e-04), and a subsequent Tukey HSD test revealed s37 to be greater than 11 isolates (s15, s16, s18, s20, s23, s24, s25, s35, s36, s39, and s41 at an adjusted *p*-value < = 0.04). Based on Mantel tests, LDH results of *B*. *cepacia* did not correlate with date of isolation (*p* = 0.27) or distances based on the SNV tree (p = 0.83). When comparing only the *B*. *contaminans* ISS-derived isolates to each other, an ANOVA of the LDH data by timepoint yielded no difference between the isolates (min *p*-value = 0.54), nor did LDH release correlate with date of isolation (*p* = 0.98; Mantel test) or distances based on the SNV tree (p = 0.21)

The macrophage intracellularization assay revealed no significant differences between ISS-derived isolates and their corresponding type-strain references (no adjusted *p*-values < 1 following Bonferonni correction; Figs 6 and 7). In comparing solely the ISS-derived isolate CFU/ml results to each other, an ANOVA of *B*. *cepacia* isolates within each timepoint yielded a difference between isolates at the 6-hour timepoint (*p* = 6e-06), and a Tukey HSD test revealed s33 to be greater than all isolates other than s28 and s31, each at an adjusted *p*-value < = 0.01. And Mantel tests revealed no correlation between CFUs and date of isolation (*p* = 0.28) or SNV tree distances (*p* = 0.81). When comparing solely the *B*. *contaminans* ISS-derived isolates against each other by timepoint via ANOVA, this returned a minimum p-value of 0.08. And again, Mantel tests revealed no correlation between date of isolation (*p* = 0.65) or distances based on SNV trees (*p* = 0.39).

The included *B*. *cepacia* ATCC25416 terrestrial reference strain has been noted for its ability to invade and carry out a long-term colonization of macrophage which can lend to the formation of long-term chronic infections [41]. However, in our assay we find that although each ISS-derived *B*. *cepacia* isolate is able to invade macrophage by 6 hours, they appear to multiply, then escape from the macrophage by 12 hours post-inoculation. Here they may be using the plasmid-encoded lysophospholipase mechanism to generate this lysis of macrophage. Despite not contributing to a longer-term infection, the cytotoxic byproducts generated by lysophospholipase degradation of macrophage may play a physiological role in further stimulating the adhesion and differentiation of lymphoid cells macrophages and activation and recruitment of additional macrophage and T-lymphocytes, among other immune response mechanisms [31]. In contrast, the *B*. *cepacia* ATCC25416 terrestrial reference strain remained intracellularized at 12-hours post-inoculation (Fig 6) while still exhibiting the ability to lyse macrophage (Fig 4). *B*. *contaminans* ISS-derived isolates and terrestrial reference strain, on the other hand, seem to exhibit a decreased rate of cellular invasion and a more immediate cell lysis of macrophage. This follows suit with the hemolytic and antifungal capabilities they also display, which is not characteristic of the *B*. *cepacia* isolates.

#### Antifungal and hemolysis assay

Non-ribosomal peptide synthetase (NRPS)-derived occidiofungin/burkholdine-like compounds are produced by *B*. *contaminans* via a gene cluster thought to have evolved to protect BCC bacteria from ecological niche predators such as amoeba and fungi [42]. Using antiSMASH [43] we verified that each of the ISS isolates and the reference strain contain the occidiofungin and pyrrolnitrin gene cluster (gene clusters presented in S6 Table). The antifungal metabolite, occidiofungin, as well as pyrrolnitrin are known to also display hemolytic properties [44], which can break down heme in the hemoglobin of host bodies causing complications for the host.

**Fig 8:**
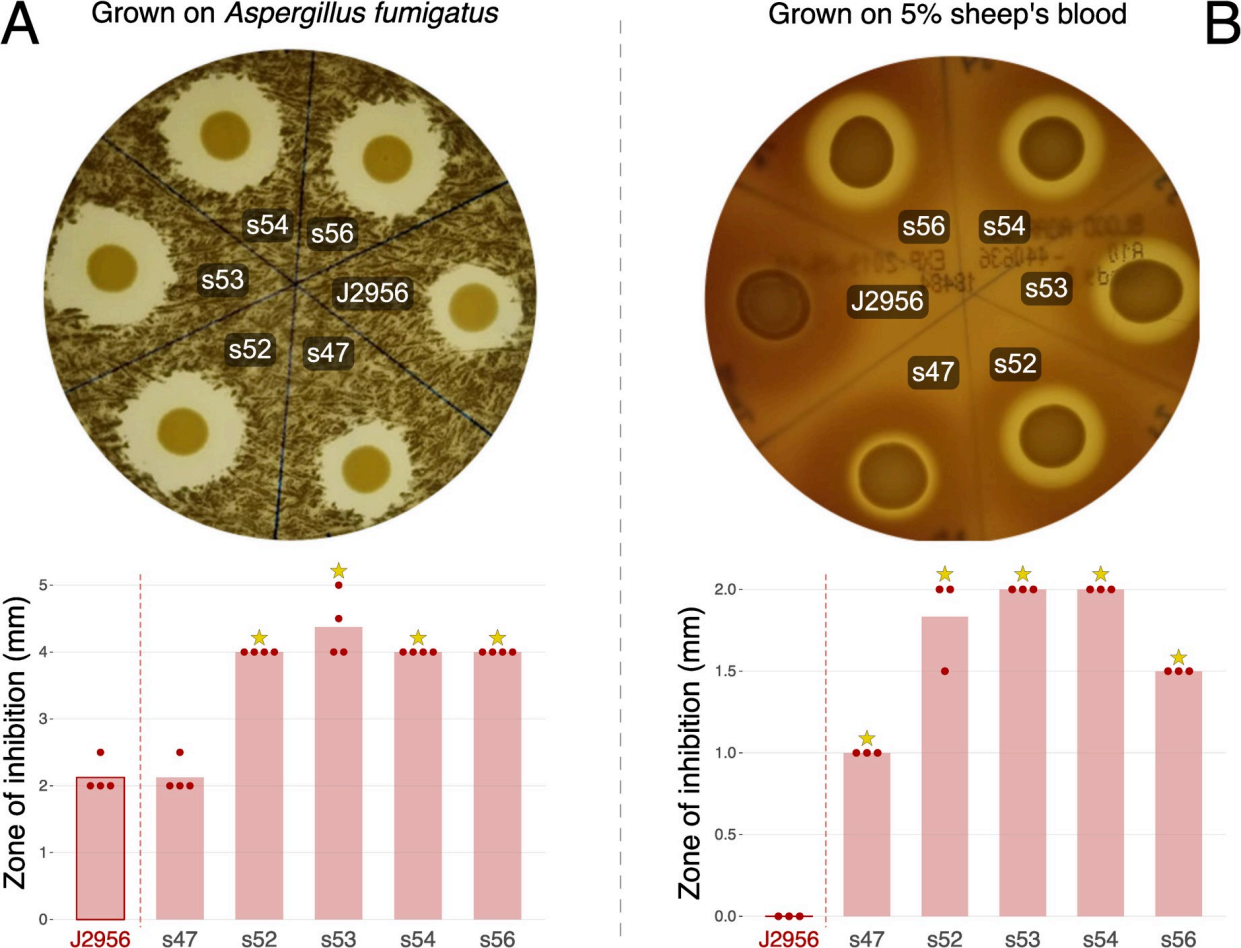
*B*. *contaminans* grown on TSB plates with *Aspergillus fumigatus* and 5% sheep’s blood. **(A)** Grown with *Aspergillus fumigatus*, n = 4. **(B)** Grown with 5% sheep’s blood, n = 3. Plotted are zones of inhibition for each in millimeters; bars represent means, all data points shown as dots. Stars indicate adjusted p-values < = 0.003 following two-sided t-tests compared to the terrestrial reference J2956 (adjusted by Bonferonni correction).

Due to the identification of *B*. *contaminans* isolates harboring these two biosynthetic gene clusters within our collection, we assayed for the ability to inhibit growth of the *Aspergillus fumigatus* AF293 strain (Fig 8A) and to cause hemolysis (Fig 8B). None of the *B*. *cepacia* isolates exhibited fungal inhibition or hemolysis, but each of the *B*. *contaminans* did to varying degrees. The reference strain (*B*. *contaminans* J2956) and ISS-isolate s47 displayed a lower amount of fungal inhibition relative to the rest of the isolates (Fig 8A; adj. *p* < = 0.003 following t-tests and Bonferonni correction). Antifungal activity did not correlate with date of isolation (*p* = 0.17) or phylogenetic distance based on SNV trees (*p* = 0.99) based on Mantel tests. In the hemolysis assay, type strain J2956 displayed little to no hemolytic activity, while all ISS-derived isolates did to some extent (Fig 8B). An ANOVA focusing on just the ISS-derived isolates (p = 1e-05), followed by a Tukey HSD test revealed differences between all and s47, as well as s53 and s54 as compared to s56 (adj. *p*-values < = 5e-03; Fig 8B). No correlation was found between date of isolation (p = 0.20) or distances based on the SNV trees (*p* = 0.67; Mantel tests).

#### Minimum inhibition concentration assay

**Fig 9:**
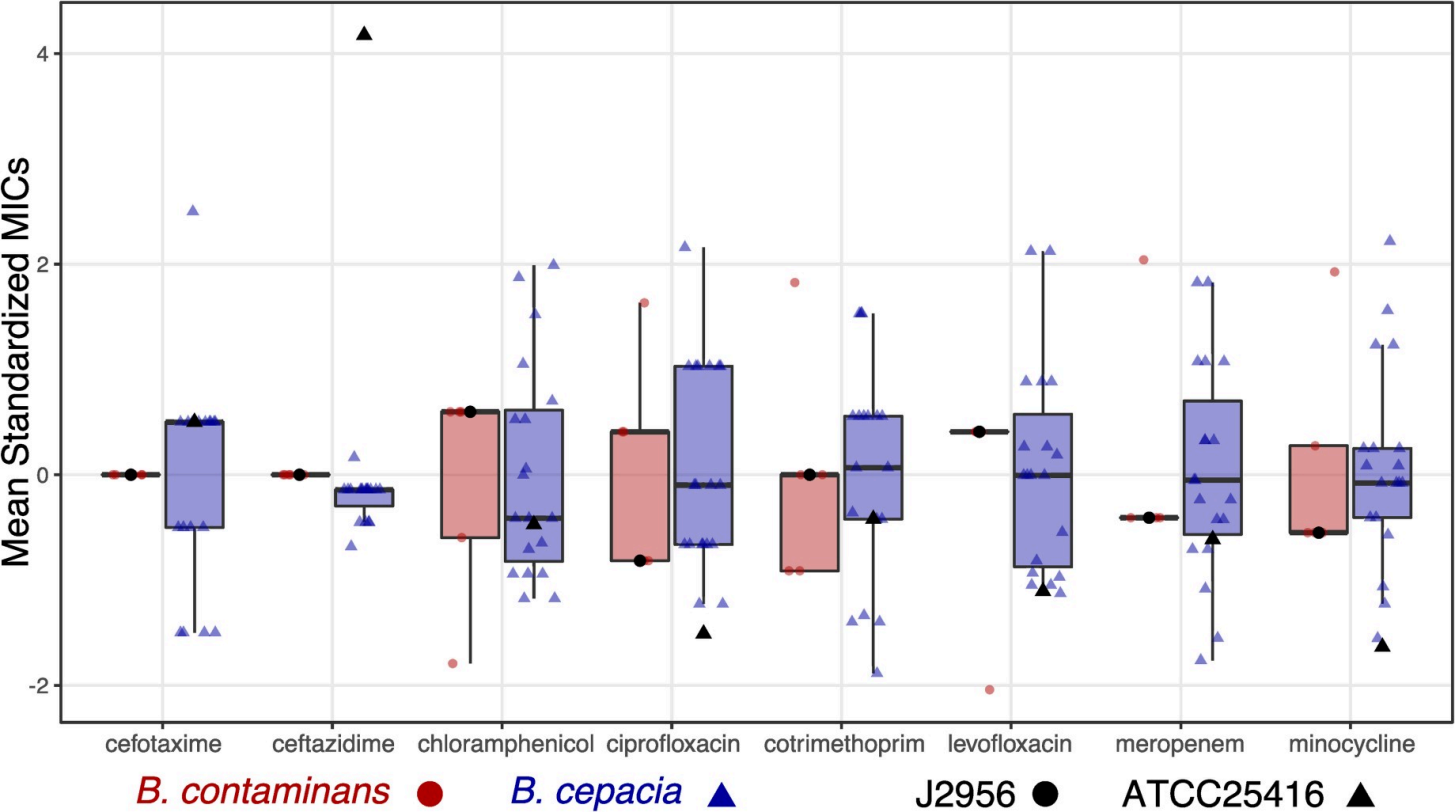
Antibiotic screening. Each individual data point plotted represents the mean of 3 replicates. Boxplots of those means standardized within that antibiotic and species are plotted for each antibiotic for both species (red and circles = *B*. *contaminans*; blue and triangles = *B*. *cepacia*). And black versions of the shapes are where the associated reference strains fall on that scale.

ISS isolates and reference isolates were assayed for resistance or susceptibility using antibiotics commonly administered in the clinical treatment of BCC infections. These antibiotics included: cefotaxime, meropenem, and ceftazidime (cell wall synthesis inhibitors); ciprofloxacin (a topoisomerase inhibitor); cotrimethoprim (trimethoprim/sulfamethoxazole- a thymidine synthesis inhibitor); chloramphenicol (a 50S ribosomal subunit inhibitor); levofloxacin (a DNA synthesis inhibitor); and minocycline (a 30S ribosomal subunit inhibitor). All isolates exhibited similar sensitivity to these antibiotics as their reference strains (minimum adj. p-value = 0.42; Fig 9; data in S7 Table; code in repository linked below), and no correlation between date of isolation and minimum inhibition concentrations was found. Focusing on comparing just the ISS-derived isolates together revealed no notable differences between the *B*. *contaminans* isolates, while for *B*. *cepacia* an ANOVA by antibiotic yielded differences for meropenem (p = 1e-04), with a follow-up TukeyHSD showing differences between a few isolates (s15-s22; s16-s33/s41; s22-s33/s36/s39/s41; s57-s33/s41; all adj. p-values < = 0.03).

#### Biofilm assay

**Fig 10:**
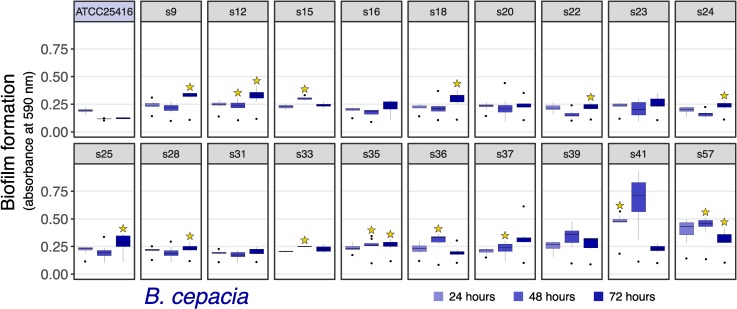
Biofilm formation of *B*. *cepacia*. Based on crystal violet assay. Boxplots of each timepoint’s absorbance values are plotted for each (n = 8 within each boxpot). Plotted are means with standard errors as error bars (n = 8). Stars indicate < 0.05 adjusted p-value from two-sided t-tests against the reference strain (adjusted by Bonferonni correction).

**Fig 11:**
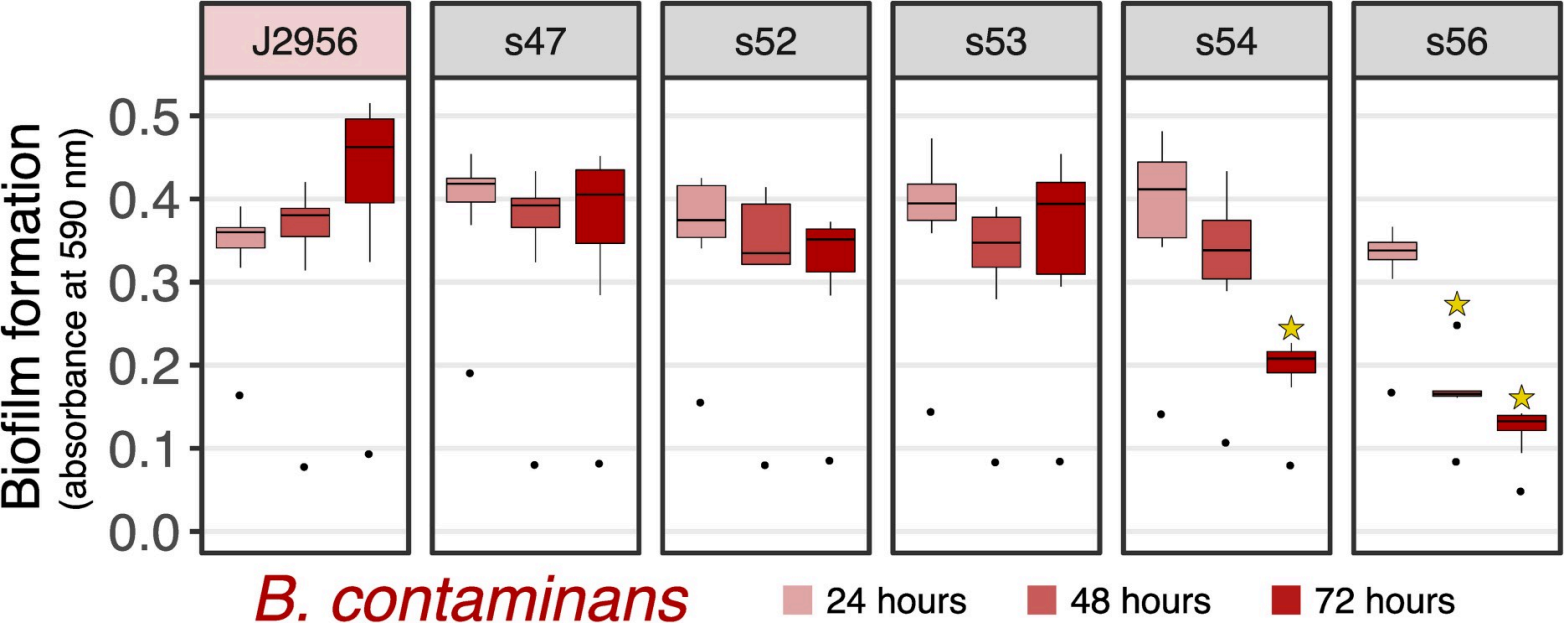
Biofilm formation of *B*. *contaminans*. Same as Fig 9. Stars indicate < 0.04 adjusted p-value from two-sided t-tests against the reference strain (adjusted by Bonferonni correction).

We observed greater biofilm formation in some of the *B*. *cepacia* isolates compared to their assayed reference strain (Fig 10). An ANOVA contrasting just the ISS-isolates by timepoint revealed differences at all timepoints (p = 4e-03), post-hoc TukeyHSD tests revealed differences between s41 and s57 compared to almost all other isolates at 24 and 48 hours (all adj. p-values < = 4e-03), and no notable differences at 72 hours (all adj. p-values > 0.05). Based on Mantel tests, *B*.*cepacia* biofilm results generally did not strongly correlate with date of isolation (p = 0.12) or phylogenetic distance based on its SNV tree (p = 0.07). *B*. *contaminans* displayed similar biofilm formation to its reference in most cases, though lower biofilm formation was observed for s54 at 72 hours, and s56 at 48 and 72 hours (all adj. p-values < = 0.04 following t. tests and bonferroni correction; Fig 11)–at least as assayed by the performed crystal violet method. Focusing on contrasting the *B*. *contaminans* ISS-isolates alone, an ANOVA by timepoint yielded differences at these timespoints as well (48 and 72 hours; p-value < 3e-03), and a follow-up TukeyHSD test revealed differences between s56 and all others at 48 hours (adj. p-value < 0.03), and differences between s56 and all (adj. p-value < 0.007), as well as s54 and s43 (adj. p-value = 0.01). Mantel tests revealed no correlation between date of isolation (p-value = 0.5) or SNV distance (p-value = 0.22).

## Conclusion

Overall, within each species, the 19 *B*. *cepacia* and 5 *B*. *contaminans* recovered from the ISS PWD over roughly a 5-year timespan were highly similar on a whole genome scale, suggesting each population (here meaning one for each species) that was sampled from may have stemmed from two distinct founding strains. This is supported by all isolates demonstrating greater than 99% ANI with 95–99.9% alignment in all cases, and sharing a core pangenome of greater than 90% of identified gene clusters (within species). The vast majority of differences in gene-content that were identified in the pangenome were localized on putative plasmids. Physiologically, including antibiotic sensitivities, the ISS-derived isolates were overall generally similar to each other, as well as to the reference type-strains analyzed–excluding a few exceptions such as *B*. *cepacia* isolates s41 and s57 and *B*. *contaminans* s54 and s56 biofilm formation rates. In cases where ISS-derived isolates did vary, no strong correlations were found between physiology and either SNV distances or date of isolation (though it is also import to keep in mind date of isolation does not necessarily indicate any information about how long an isolate had been aboard the ISS). While it is interesting the 5 recovered *B*. *contaminans* isolates were all isolated in a span of time over 2013 and 2014 during which no *B*. *cepacia* were isolated (see Fig 1A), we cannot speak to what may have caused this at this time (if anything). That aside, our analysis here suggests these two species’ populations sampled by way of isolation seem to be highly similar genetically regardless of date of isolation, with the majority of variation that does exist being maintained among putative plasmids. Based on the information presented here, it seems likely that the two populations of *Burkholderia* present in the ISS PWS are not more virulent than those that might be encountered on planet.

## Methods

### DNA isolation

Bacterial isolates from the ISS PWD were obtained by filtering sample from both the ambient and hot water outlets of the potable water system through a microbial capture device (MCD) on the ISS by resident astronauts. The MCD filter was then placed on R2A agar and the plates were sent back to Earth for further isolation at JSC. Additionally, 1 liter of water was sent back in to Earth for further isolation at JSC on R2A agar. All isolates were cataloged and then stored at -80C in 15% glycerol until regrown on R2A agar slants and sent to JCVI. No permits were required for this transport. JCVI conducted an internal review to ensure safe handling and containment. Upon arrival at JCVI all isolates were further plated on tryptic soy agar (TSA) and incubated overnight at 35°C, then inoculated into 4mls of tryptic soy broth (TSB). 2mls were stored with 15% glycerol as 1ml bacterial stocks and 2mls were centrifuged to form pellets with supernatant decanted then immediately frozen at -80C for future DNA extraction. DNA was extracted using a standard phenol/chloroform protocol. DNA was quantified using nanodrop and run on a gel to ensure high molecular weight samples were obtained.

### Genomic library preparation and sequencing

400ng of high molecular weight bacterial in 13ul of 1X TE (10mM Tris pH 8.0, 1mM EDTA) was processed using the NEBNext Ultra II FS DNA Library Prep Kit for Illumina (New England Biolabs, Ipswich, MA) protocol at half the standard reaction volumes. To the 13ul DNA, 3.5 ul of NEBNext Ultra II FS Reaction Buffer and 1ul of NEBNext Ultra II FS Enzyme was added in a PCR tube and then incubated in a thermocycler at 37°C for 10 minutes at 37°C for fragmentation to target size, the enzyme is then denatured by incubating the reaction at 65°C for 30 minutes with lid set to 75°C. Next, adapter ligation is carried out on the 17.5 ul mixture of fragmented DNA by adding 15 ul of NEBNext Ultra II Ligation Master Mix, 0.5 ul of NEBNext Ligation Enhancer, 1.25 ul of NEBNext Adapter for Illumina for a total volume of 34.25 ul. The 34.25 ul adapter mixture is incubated at 20°C for 15 minutes a thermocycler with the heated lid off. This is followed by the addition of 1.5ul of USER Enzyme to the mixture, then incubation in the thermocycler at 37C for 15 minutes with heated lit set to 47C. In order to obtain 700–900 bp inserts, we used SPRIselect beads for a rightside clean-up of 0.25X, where the supernatant is retained for a left side clean up using a 0.25X bead clean up then eluted in 7.5 ul of 0.1X TE buffer. The fragmented, adapter ligated and size selected libraries were then amplified using 12.5 ul of NEBNext Ultra II Q5 Master Mix with 2.5 ul i7 index primer and 2.5 ul i5 Universal PCR primer for a total volume of 25 ul. The amplification was carried out at the following temperatures and times: initial denaturation at 98°C for 30 seconds, followed by 4 cycles of denaturation at 98°C for 10 seconds and annealing/extension at 65°C for 75 seconds, and a final extension at 65°C for 5 minutes. A total of 58 libraries were generated, quality controlled to find library size using the Agilent Bioanalyzer and high sensitivity DNA chip and double stranded DNA concentration was quantified using Qubit Fluorometric Quantitation. Libraries were normalized to achieve a total of 700 pM of pooled library in 200 ul with an average library size of 750 bps. An average of 7 million, 150 bp reads were obtained for the combined read 1 and read 2 of each library using Illumina’s NextSeq 500 High Output Kit.

### Genomic sequencing postprocessing

For the 24 libraries, demultiplexing was carried out allowing 1 bp mismatch and adapters were trimmed. bcl2fastq2 Conversion Software v2.17 was used to check for adapters, if detected, base calls matching the adapter and beyond the match are masked or removed in the resultant FASTQ file. Sequence quality was assessed using the program FastQC [45] Genomic DNA library forward and reverse reads were quality trimmed using trimmomatic v0.39 [46] with a sliding window of 5:20 and a minimum length of 100 base pairs. Libraries were de novo assembled using SPAdes v3.12.0 [16]. Coverage values for assembled genomes ranged from 31–832 (143.7 ± 153; mean ± 1SD), and additional assembly summary information is presented in S1 Table. Genomes were annotated with NCBI’s Prokaryotic Genome Annotation Pipeline [47] as well as with NCBI COGs [48] within anvi’o v5.5 [23]. Average nucleotide identity was calculated with fastANI v1.2 [17]. An estimated maximum-likelihood tree made with GToTree v1.4.4 [20], based on amino acid sequences of 203 single-copy genes specific to Betaproteobacteria (see GToTree) using FastTree2 v2.1.10 [49]. Whole-genome assembly single-nucleotide variant (SNV) trees were generated with Parsnp v1.2 [21].

All pangenomic analyses were performed within anvi’o v5.5 [23], which uses MCL clustering [22]. Default settings were used other than setting the `—mcl-inflation`parameter to 7. All processing and analysis code (command-line and within R) are available at the link below under “Data availability”.

### Functional enrichment/depletion analysis

This was performed within anvi’o v5.5 [23] utilizing the `anvi-get-enriched-functions-per-pan-group`program with default settings. The resulting table was filtered to include only those with a Benjamini-Hochgerg corrected p-values of < = 0.1.

### Macrophage infection

The infection assay aimed for a multiplicity of infection (MOI) of 5 ISS *B*. *cepacia* and *B*. *contaminans* cells for every one macrophage cell. Biological replicates of the experiment were conducted on three separate days to ensure a robust reproducibility in results. At time zero CFU/mL count was conducted for inoculum of each ISS isolate and was averaged for each biological replicate experiment of the separate days. These counts showed that our experimental MOI was in the range of MOI 5–11 with an average of MOI of 7 over the three replicated experiments. The bacteria were allowed to infect the macrophage cells for two hours before being washed away and replaced with fresh media containing 15ug/mL ceftazidime and 1mg/ml amikacin to ensure clearance of extra cellular bacteria. At six, eight, twelve and 24 hours post-infection, the ISS treated sample supernatant and control supernatant from uninfected cells is collected and macrophage cells were removed by centrifugation at 300rpm for 2mins. The supernatant was then assessed for the presence of lactase dehydrogenase (LDH) using a Cytotoxicity Detection Kit (LDH) (Roche, Germany). The cell-free supernatant is incubated with the reaction mixture from the kit. LDH activity is determined using an enzymatic test. In the first step NAD+ is reduced to NADH/H+ by the LDH-catalyzed conversion of lactate to pyruvate. In the second step the catalyst (diaphorase) transfers H/H+ from NADH/H+ to the tetrazolium salt INT which is reduced to formazan. The enzyme reaction was stopped by the addition of 50 l/ well 1N HCl (final concentration: 0.2 N HCl) after 10 minutes of incubation. The formazan dye absorbance was measured using the 490nm wavelength. We present the LDH data for all ISS strains and *B*. *contaminans* J2956 and *B*. *cepacia* ATCC25416 reference strains (Figs 4 and 5; n = 3). The no-infection control was no different than the wells with media alone where no LDH was released due to a lack of cells. The media and cells plus media alone were similar for all timepoints. The cells were growing well and not dying due to our selected seeding number at the start of the experiment. The cells plus media control or the “no-infection control” and media control absorbance values were averaged and subtracted from the treatments as background values.

Additionally, the macrophages were washed after removal of the supernatants at six, eight, twelve- and 24-hours post-infection to remove any dead extracellular bacterial cells, then lysed in order enumerate the intracellular bacterial cells by plating and colony counting. For this and the following physiology reported, t-tests were performed followed by Bonferroni correction in comparing the ISS-derived isolates to the type-strain references, and ANOVA (followed by a post-hoc TukeyHSD test when appropriate) was performed to compare the isolates. Mantel tests were performed to check for correlations between physiology and date of isolation and distance based on the SNV trees. All R code linked below.

### Aspergillus fumigatus screen

JCVI -80°C freezer stocks were streaked onto TSA and incubated 48 hours at 37°C, single colonies were then inoculated into 3 mLs TSB and incubated overnight at 37°C with shaking at 220rpm. *Aspergillus fumigatus* AF293 strain was maintained as glycerol stocks and grown at 37°C. The fungus was incubated on Potato Dextrose Agar (PDA; Acumedia Manufactures Inc. Lansing, MI) at 37°C under the dark for 4 days. The fungal spores were collected with loops and suspended with 0.01% tween 80 sterilized water. The spore suspension was counted by hemocytometer and was inoculated to 10^6 spores per plate to PDA and TSA media, concurrently 5 μl of a *Burkholderia* isolate was spotted in a quadrant of each of the agar types. The samples were incubated at 37°C and the zones of inhibition were recorded after 48hours. Zone of inhibition sizes were achieved by measuring the diameter of the outer clearance zone and then subtracting the diameter of the bacterial growth zone, and dividing by 2. N = 4.

### Hemolysis assay

JCVI -80°C freezer stocks were streaked onto TSA and incubated 48 hours at 37°C, single colonies were then inoculated into 3 mLs TSB and incubated overnight at 37°C with shaking at 220 rpm. 5 μl of each *Burkholderia* isolate culture was spotted onto a petri plate (100mm x 15mm) containing TSA with 5% sheep's blood and incubated at 37°C for 48 hours. N = 3.

### MIC determination

1xMICs of all antibiotics were determined using the National Committee for Clinical Laboratory Standards (NCCLS). In the MIC assay, 2ul of an antibiotic dilution series (beginning at 64ug/mL) was used to treat 100ul of OD_600_ = 0.001 culture (diluted from cells in exponential growth phase), then incubated without shaking for 18 hours before MIC determination. The MIC value is the lowest concentration at which bacterial growth is fully arrested. N = 3.

### Biofilm assay

JCVI -80°C freezer stocks were streaked onto TSA and incubated 48 hours at 35°C, single colonies were then inoculated into 3 mLs TSB and incubated overnight at 3°C with shaking at 220 rpm. Cultures were diluted to an OD600 of 1, then diluted 1:100, each culture was then plated into one column of three 96 well polystyrene plates at 150 μl per well. The cultures were covered with adhesive film, then incubated at 35°C for 24, 48, and 72 hours. At each timepoint an entire 96 well plate having 11 cultures and control culture were stained using crystal violet (3g/L solution). This process includes removing the cultured media from each well by inverting the plate and giving it 3–5 abrupt shakes into a waste container, then rinsing the wells lightly with water. This is followed by the addition of 150 μl of crystal violet into each well, the crystal violet is incubated in the well to adhere to any remaining biofilm for exactly 15 minutes, then the plate is carefully inverted and given 3–5 abrupt shakes into a crystal violet waste container. The plate is carefully rinsed with water and then laid upside down to dry. Once each of the 3 time points have been processed, the crystal violet is resolubilized for each timepoint plate using 100% EtOH by pipette mixing, then transferred to a new plate and covered to avoid evaporation. The resolubilized crystal violet strain is then placed in a plate reader to obtain an absorbance at 590 nm. N = 8.

## Supporting information

S1 Fig ISS-derived *Burkholderia* isolates compared with 20 *B*. *contaminans* and 136 *B*. *cepacia* from NCBI as accessed on 15-Aug-2019. An estimated maximum-likelihood phylogenomic tree based on aligned and concatenated amino-acid sequences of 203 single-copy genes designed for targeting Betaproteobacteria placed 19 ISS *B*. *cepacia* isolates and 5 ISS *B*. *contaminans* isolates within their own monophyletic clades. Rooted with *Ralstonia pickettii* 12J (GCF_000020205.1).
(TIF)

S2 Fig Clustering based on Gene Clusters (GCs) and Single-nucleotide variant (SNV) trees for *B*. *cepacia*. Clustering based on GCs did not recapitulate the SNV trees for *B*. *cepacia*.
(TIF)

S3 Fig Clustering based on Gene Clusters (GCs) and Single-nucleotide variant (SNV) trees for *B*. *contaminans*. Clustering based on GCs did not recapitulate the SNV trees for *B*. *contaminans*.
(TIF)

S1 Table Isolate information and assembly summary data. Sample ID, NASA isolate ID, Mission and date isolation and *de novo* assembly summary information.
(TSV)

S2 Table *B*. *contaminans* functional enrichments. Functional annotations of the gene enrichments for 5 ISS-derived *B*. *contaminans* and 20 references.
(TSV)

S3 Table *B*. *cepacia* functional enrichments. Functional annotations of the gene enrichments for 19 ISS-derived *B*. *cepacia* and 136 references.
(TSV)

S4 Table ISS-isolate-only pangenomes with annotations and AA sequences. Gene calls, amino acid sequences, and annotations.
(TSV)

S5 Table ISS-isolate-only plasmid pangenomes with annotations and AA sequences. Gene calls, amino acid sequences, and annotations for putative plasmids.
(TSV)

S6 Table Gene IDs for predicted occidiofungin and pyrrolnitrin clusters in *B*. *contaminans*. Gene IDs of the identified occidiofungin and pyrrolnitrin clusters for isolates s47, s52, s53, s54, and s56.
(TSV)

S7 Table Antibiotic screening MIC data. MICs for all ISS *Burkholderia* isolates for the antibiotics: cefotaxime, meropenem, and ceftazidime (cell wall synthesis inhibitors); ciprofloxacin (a topoisomerase inhibitor); cotrimethoprim (trimethoprim/sulfamethoxazole- a thymidine synthesis inhibitor); chloramphenicol (a 50S ribosomal subunit inhibitor); levofloxacin (a DNA synthesis inhibitor); and minocycline (a 30S ribosomal subunit inhibitor).
(TSV)
