# Supplementary figures and images for: Genomic and phenotypic characterization of Burkholderia isolates from the potable water system of the International Space Station
Source: PLoS One. 2020 Feb 19;15(2):e0227152. doi: 10.1371/journal.pone.0227152 (PMC7029842; doi:10.1371/journal.pone.0227152)

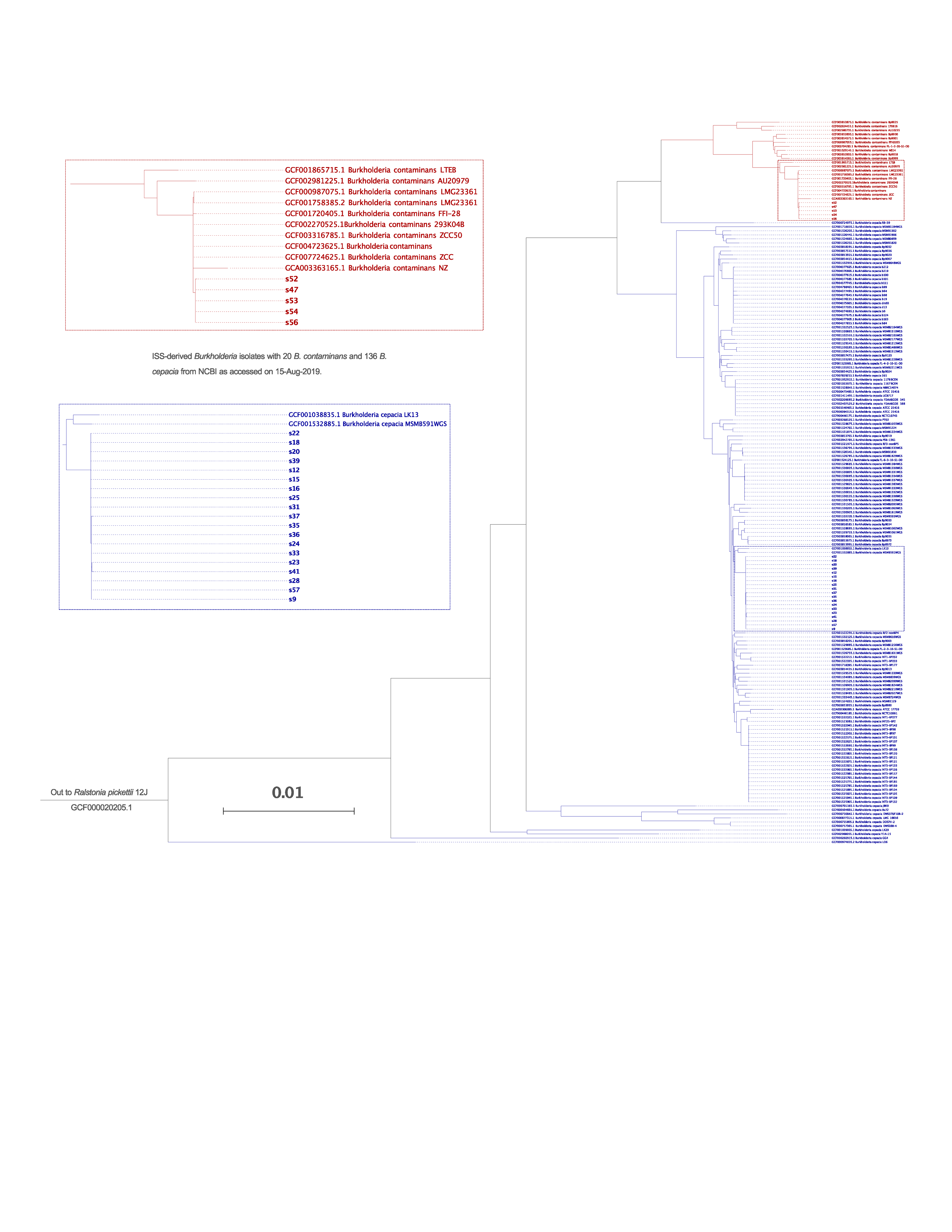

Supplement: S1 Fig — An estimated maximum-likelihood phylogenomic tree based on aligned and concatenated amino-acid sequences of 203 single-copy genes designed for targeting Betaproteobacteria placed 19 ISS B. cepacia isolates and 5 ISS B. contaminans isolates within their own monophyletic clades. Rooted with Ralstonia pickettii 12J (GCF_000020205.1). (TIF) [file pone.0227152.s001.tif]

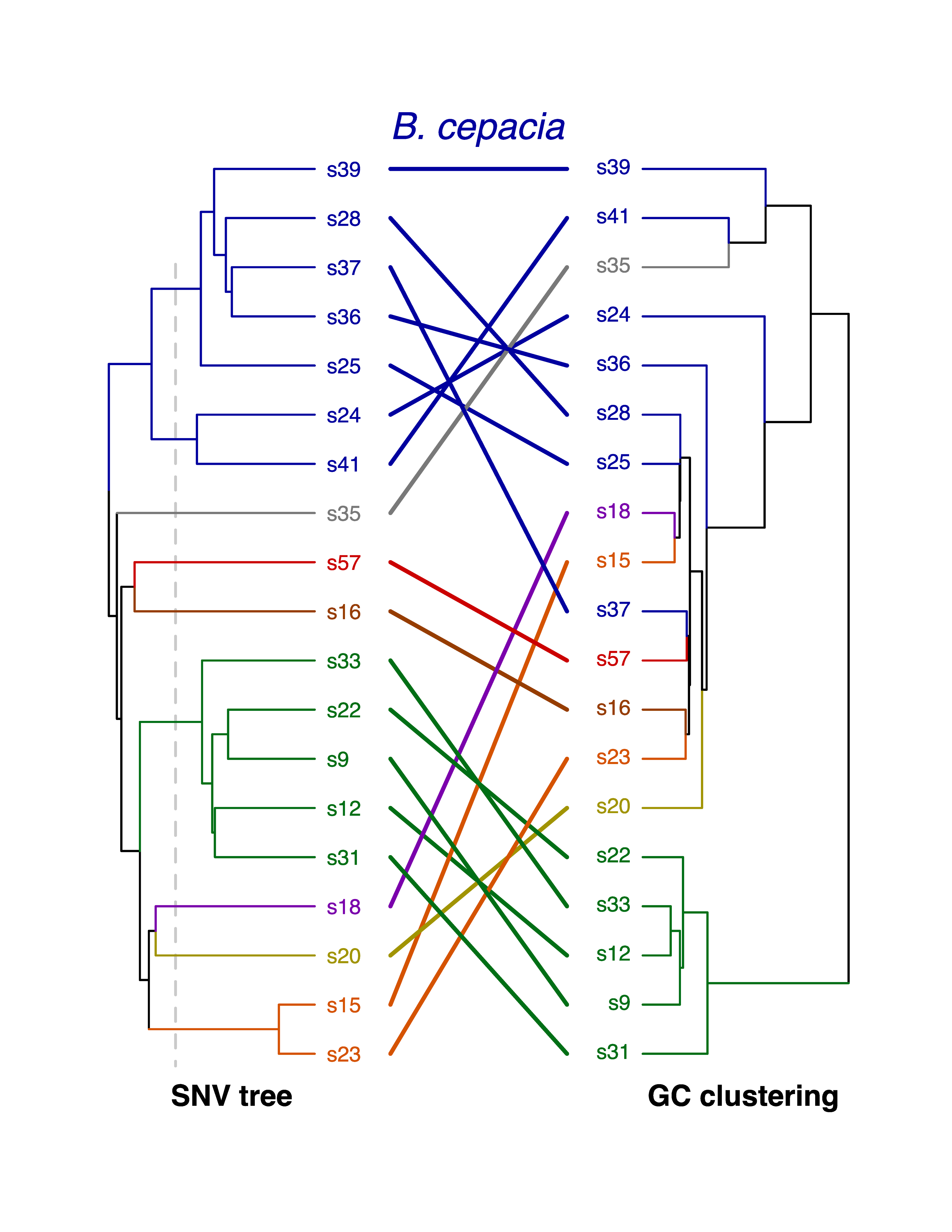

Supplement: S2 Fig — Clustering based on GCs did not recapitulate the SNV trees for B. cepacia. (TIF) [file pone.0227152.s002.tif]

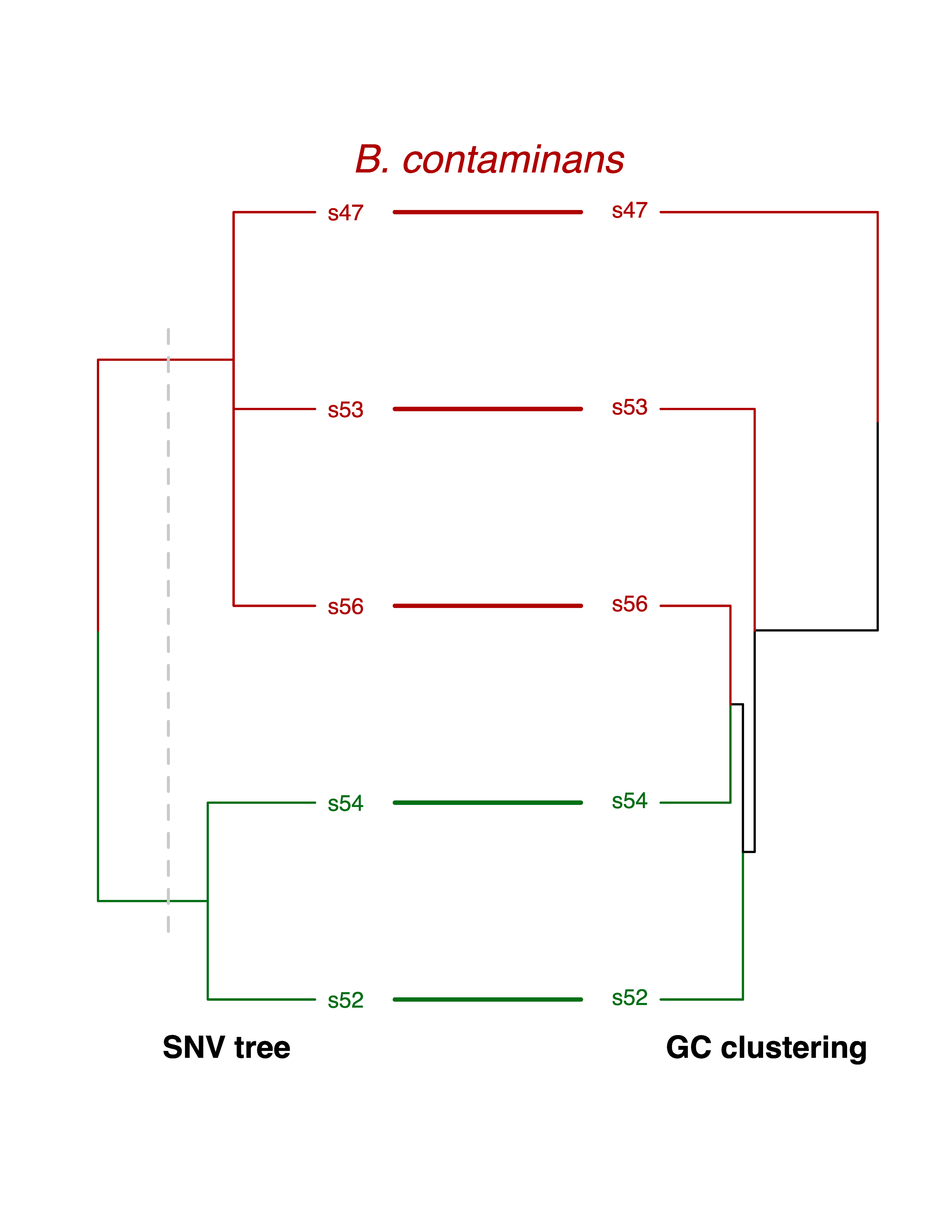

Supplement: S3 Fig — Clustering based on GCs did not recapitulate the SNV trees for B. contaminans. (TIF) [file pone.0227152.s003.tif]
